# Investigation into the Individualized Treatment of Traditional Chinese Medicine through a Series of N-of-1 Trials

**DOI:** 10.1155/2018/5813767

**Published:** 2018-02-07

**Authors:** Haiyin Huang, Peilan Yang, Jie Wang, Yingen Wu, Suna Zi, Jie Tang, Zhenwei Wang, Ying Ma, Yuqing Zhang

**Affiliations:** ^1^Department of Respiratory Disease and Department of Pharmacy, Yueyang Hospital of Integrated Traditional Chinese and Western Medicine, Shanghai University of Traditional Chinese Medicine, Shanghai 200437, China; ^2^Longhua Hospital, Shanghai University of Traditional Chinese Medicine, Shanghai 200032, China; ^3^Guang'anmen Hospital, China Academy of Chinese Medical Science, Xicheng District, Beijing, China; ^4^Department of Health Research Methods, Evidence, and Impact, McMaster University, Hamilton, ON, Canada L8S 4K1

## Abstract

**Purpose:**

To compare the efficacy of individualized herbal decoction with standard decoction for patients with stable bronchiectasis through N-of-1 trials.

**Methods:**

We conducted a single center N-of-1 trials in 17 patients with stable bronchiectasis. Each N-of-1 trial contains three cycles. Each cycle is divided into two 4-week intervention including individualized decoction and fixed decoction (control). The primary outcome was patient self-reported symptoms scores on a 1–7 point Likert scale. Secondary outcomes were 24-hour sputum volume and CAT scores.

**Results:**

Among 14 completed trials, five showed that the individualized decoction was statistically better than the control decoction on symptom scores (*P* < 0.05) but was not clinically significant. The group data of all the trials showed that individualized decoction was superior to control decoction on symptom scores (2.13 ± 0.58 versus 2.30 ± 0.65, *P* = 0.002, mean difference and 95% CI: 0.18 (0.10, 0.25)), 24 h sputum volume (*P* = 0.009), and CAT scores (9.69 ± 4.89 versus 11.64 ± 5.59, *P* = 0.013, mean difference and 95% CI: 1.95 (1.04, 2.86)) but not clinically significant.

**Conclusion:**

Optimizing the combined analysis of individual and group data and the improvement of statistical models may make contribution in establishing a method of evaluating clinical efficacy in line with the characteristics of traditional Chinese medicine individual diagnosis and treatment.

## 1. Introduction

Randomized controlled trials (RCTs) have been widely recognized as the best research design in evaluating the efficacy and safety of medical intervention. However, in clinical practice, especially for the treatment of chronic diseases, the effective interventions validated in population-based randomized controlled trials may be invalid for some individual cases due to great individual differences [1]. Therefore, the ability to apply findings from population-based RCTs in clinical practice may be diminished, especially in traditional Chinese medicine (TCM) which emphasises individualized treatment [2]. TCM is one of the main complementary and alternative medicine modalities around the world, and it plays an increasingly important role in international medical practice [3]. Individualized treatment based on individual patient syndrome differentiation guided by a “holistic view” is one of the characteristics of TCM. This individualized TCM intervention often makes it difficult for population-based RCTs to carry out an accurately representative standard* format*. The use of herbal decoctions with fixed herbs or patent Chinese herbal medicine may not represent adequately the individual clinical efficacy that TCM is argued to be capable of with regular clinical use of individualized herbal prescriptions and treatments. RCTs of population-based treatments cannot therefore adequately assess the clinical efficacy of TCM. The lack of a reliable and evidence-based method of evaluating the clinical efficacy of TCM has hindered its internationalization and its further development [2]. It is therefore important to attempt to establish a method of evaluating clinical efficacy which is consistent with the characteristics of TCM individual diagnosis and treatment [4].

Single case randomized controlled trials (N-of-1 randomized controlled trials, referred to as N-of-1 trials) take the subject him/herself as the control and the result eventually applied to guide the treatment of the subject him/herself. The sample size of the trial is 1. The idea of individualized treatment represented by N-of-1 trials coincides naturally with the principle of (necessarily individually applied) syndrome differentiation of TCM and provides a feasible way for the linking up between TCM and western medicine. Therefore it is conducive to the integrity of this linkage to carry out the research on the efficacy of traditional Chinese medicine by N-of-1 trials [2, 5].

Bronchiectasis is a chronic respiratory disease characterized by a clinical syndrome of cough, sputum production, and bronchial infection, and radiologically by abnormal and permanent dilatation of the bronchi. According to the guidelines for adult bronchiectasis by European Respiratory Society, the prevalence of bronchiectasis has been estimated at 53 to 566 cases per 100,000 inhabitants. Prevalence increases with age and female gender [6]. In China, bronchiectasis is one of the common chronic respiratory diseases [7]. A cross-sectional study conducted in China including 10811 participants age over 40 showed a 1.2% prevalence (135/10811) of bronchiectasis [8]. However, treatment regimens are not well defined and patients tend to have ongoing symptoms even in the stable stage [7]. TCM plays an important role in the management of bronchiectasis in China. There is no standard traditional Chinese herbal decoction for stable bronchiectasis. The principles of treatment are based on TCM syndrome differentiation including reducing phlegm, clearing the lung heat, and strengthening healthy energy [9]. According to TCM theory, efficacy can only be obtained through syndrome differentiation and treatment. Stable bronchiectasis is a good indication for conducting N-of-1 trials: (1) not self-limited disease, (2) relatively stable condition, and (3) the treatment needs to be long term [1, 10]. However, N-of-1 trials require that the treatment or treatments must have a rapid onset and termination of action, and an optimal treatment duration should be known and practical [1, 10]. Whether TCM treatment can meet this requirement is still unknown and worthy of our exploration.

In the previous study, we confirmed the feasibility of conducting the study of traditional Chinese herbal decoction through N-of-1 trials [11]. Based on the results from that study, we prolonged the length of the observation period to 4 weeks and expanded the sample size in the present study. By comparing the efficacy of individualized herbal decoction (based on TCM syndrome differentiation) with controlled decoction (based on disease differentiation) for individual patients with stable bronchiectasis, we explored the possibility of using N-of-1 trials to evaluate the clinical efficacy of TCM individual diagnosis and treatment.

## 2. Methods

### 2.1. Study Design

These N-of-1 trials were randomized, double-blind, crossover comparisons of individualized herbal decoction with control decoction within individual patients. N-of-1 trials were offered to the patients meeting the inclusion criteria and who had shown a clinical response to TCM in an open preliminary trial.

Each N-of-1 trial consisted of three cycles with treatment and control assigned in random order. If acute exacerbation of bronchiectasis occurred, antibiotics and other treatments were provided conventionally [7]. We resumed the study when infection was controlled and the disease returned to stable stage.

### 2.2. The Evaluation of Washout Period and the Determination of Observation Period

**Figure 1:**
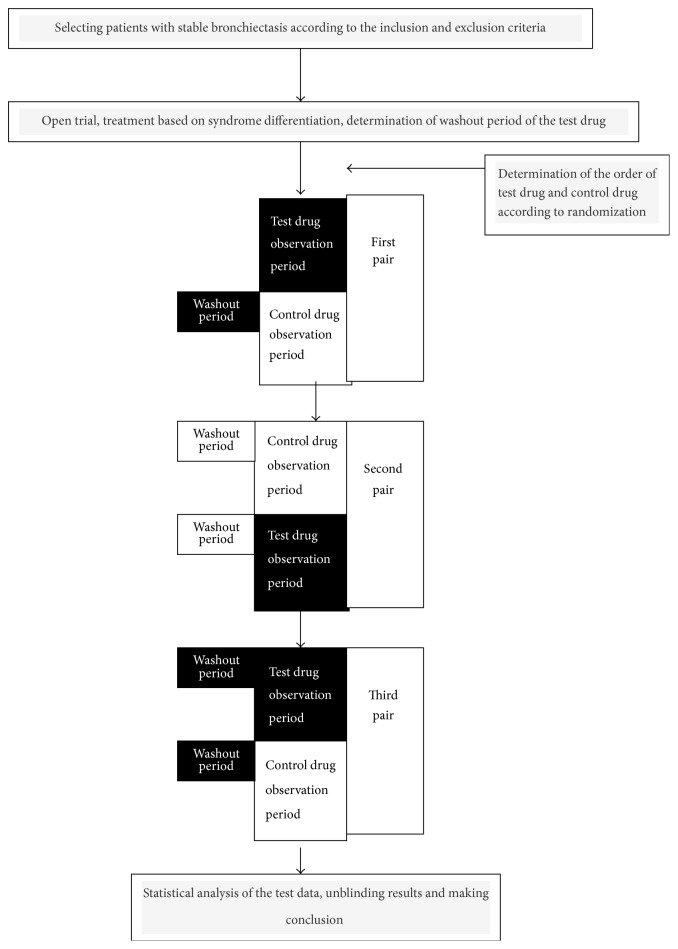
The flow chart of the N-of-1 trials in the treatment of stable bronchiectasis by traditional Chinese medicine based on syndrome differentiation.

As herbal decoction is a mixture of herbs, it is difficult to determine the half-life period biochemically. The relatively reasonable washout period can only be determined on the basis of past treatment experience and preliminary trial. The open-label preliminary trial was carried out according to the literature [11]. With changes in patients self-rated symptom scores as the main outcomes, preliminary trials can obtain onset time after drug administration and efficacy maintenance time after drug withdrawal, so as to determine the observation period and the washout period. We measured outcomes in the last week of each observation period, and the time before this was supposed to be the washout period (Figure 1).

After the preliminary trials, the observation periods of Case 1 and Case 2 were fixed to two weeks each, and case 3 three weeks. Referring to the previous results [11], we fixed the observation periods of other cases to four weeks to extend the washout periods to three weeks.

### 2.3. Patients and Diagnosis

Outpatients were eligible if they meet the following criteria: (1) the diagnostic criteria based on the consensus of Chinese experts [7] and the guidelines for noncystic fibrosis bronchiectasis issued by the British Thoracic Society in 2010 [12]; (2) male or female, aged 18–75 years; (3) being in the stable stage, and no acute exacerbation of bronchiectasis within the past three weeks; (4) frequency of acute exacerbation of bronchiectasis ≤3 times every year; (5) signed informed consent for participation. The exclusion criteria include (1) having developed respiratory failure with estimated survival time less than one year; (2) having hemoptysis as a comorbidity; (3) having complications by active tuberculosis; (4) being pregnant or with severe heart, liver, and kidney dysfunctions; (5) participating in other pharmacological clinical trials within the past 3 months.

TCM syndrome differentiation diagnostic criteria were based on the “Criteria of Diagnosis and Therapeutic Effect of TCM Diseases” issued by the State Administration of Traditional Chinese Medicine [13] and integrated with the TCM differentiation of bronchiectasis summarized from the literature [14], mainly including lung and spleen deficiency syndrome, qi and yin deficiency syndrome, and phlegm-heat obstructing lung syndrome (including mild phlegm-heat syndrome). Patients with corresponding two primary symptoms or more than two accompanied symptoms with the corresponding tongue and pulse signs could be diagnosed as having the TCM syndrome.

Syndromes of each patient were independently assessed by two associate physicians (or higher title physicians). If there was any controversy, it was decided by a third party (a distinguished veteran doctor of TCM).

### 2.4. Randomization and Blinding

We used block randomization and the block size was 2 [11]. Random numbers and random number sequence were generated by a pharmacist by computer (software SPSS 15.0) to determine the order of administration for each observation period in each single case, such as BA-AB-BA or AB-BA-BA. Doctors prescribed both individualized prescription and control prescription after assessing patients' TCM syndrome. Then the two prescriptions together with the randomized medication order were delivered to a pharmacist specifically designated by the TCM Pharmacy. The pharmacist used the coin tossing method to determine which one of A or B represented individualized prescription or control prescription, recorded the blind code, and put it for safe keeping. Then the pharmacist prepared the herbs of the prescription following the randomized medication order. The decoction of TCM was made in the decoction room of our hospital and dispensed to the patient. This method successfully kept the doctor blinded during the contact between doctors and the pharmacist. The test drug and control drug had no differences in dosage form, appearance, color, specification, label, and so forth. Doctors, patients, and outcome assessors were all blinded. Because of the unique perception, taste, and smell of traditional Chinese medicine, it is extremely difficult to find a control drug completely consistent with the test drug [15]. In our study the two TCM decoctions could be similar in appearance and size, but the slight difference in taste and smell may still exist. In order to compensate for this difference, we told the participants that both the test and control decoctions could be effective regardless of the taste and smell. Even if there is the difference, most participants were not aware which type of formula they were assigned to, as they did not show preference towards certain decoction.

### 2.5. Interventions

Basic treatment was chest physical therapy, mainly, including postural drainage and chest percussion to help expel the sputum. If acute exacerbation of bronchiectasis occurred, antibiotics and other treatments were provided [7, 12]. Concomitant treatments were used at the same time for other chronic diseases such as hypertension, coronary heart disease, and diabetes, but the usage was relatively fixed. Detailed medication records were made.

*(A) Bronchiectasis Stabilization Decoction (Control Decoction, CD) Applied in the Control Drug Observation Period*. This decoction (CD) contains eight herbs [9, 11]: Radix Lithospermi 15 g, Rhizoma Fagopyri Cymosi 30 g, Radix Ophiopogonis 15 g, Poria 15 g, Astragalus Astragali 20 g, Rhizoma Bletillae 10 g, Platycodon grandiflorum 10 g, and Semen Coicis 30 g.

*(B) Syndrome Differentiation Decoction (Individualized Decoction, ID) Applied in the Tested Drug Observation Period*. ID was the modification of CD based on syndrome differentiation. Treatment based on syndrome differentiation is the prime intervention of TCM in clinical practice. In this study, we prescribed the individualized decoction based on syndrome differentiation, together with the accompanying symptoms of each patient. For example, for patients with lung and spleen qi deficiency syndrome, we added Radix Codonopsis Pilosulae, Pericarpium Citri Reticulatae, and Atractylodes Macrocephala Koidz; for patients with qi and yin deficiency syndrome, we added Radix Adenophorae, Radix Glehniae, and Radix Rehmanniae; for patients with obvious phlegm-heat syndrome, we added Radix Scutellariae and Herba Violae. The herbs in a prescription changed according to different symptoms of individual patients. We added Radix Asteris, Flos Farfarae, and Periostracum Cicadae for frequent cough. For patients who had excessive phlegm, we added herbs such as Semen Benincasae, Rhizoma Pinelliae, Bulbus Fritillariae Thunbergii, Bulbus Fritillariae Cirrhosae. For patients with chest pain, we added Rhizoma Corydalis, Pollen Typhae and Radix Bupleuri. When asthma was seen, Fructus Perillae, Semen Armeniacae Amarum, and Periostracum Cicadae were added. Radix Codonopsis Pilosulae and Herba Agrimoniae were added for fatigue. We added Pericarpium Citri Reticulatae, Radix Saussureae, and Fructus Amomi for loss of appetite. We added Bulbus Lilii, Rhizoma Acori Graminei, and Spica Prunellae for insomnia. Besides, we also adjusted the individualized decoction in accordance with the change in the patient's condition throughout the whole study duration, whereas the fixed decoction (the same for all patients in the control group) remained unchanged. Some examples included case 2, case 3, and case 7.

*Case 2*. Female, aged 57 years, had cough, large volume of yellow purulent sputum about 70 mL a day, with occasional wheezing, good appetite, good sleep, and normal urine and stool. In recent years, the patient visited many doctors, but the efficacy was not satisfactory. Before treatment, liver and kidney functions were normal. Chest CT: bronchiectases in two lungs were complicated by infection and emphysema, and multiple bullae in the left lower lobe. The tongue was red, coating was yellow and greasy, and the pulse was slippery. TCM syndrome differentiation was diagnosed as phlegm-heat storing in lung accompanied by qi and yin insufficiency.* Treatment rule* was to clear the lung heat and reduce phlegm combined with nourishing qi and yin.* Syndrome differentiation (individualized) decoction* is as follows: Radix Scutellariae 30 g, Herba Violae 30 g, Rhizoma Fagopyri Cymosi 30 g, Platycodon grandiflorum 10 g, Semen Coicis 30 g, Semen Benincasae 30 g, Poria 15 g, Astragalus Astragali 15 g, Radix Asteris 15 g, Radix Adenophorae 15 g, Radix Ophiopogonis 15 g, Radix Glycyrrhizae 5 g, Fructus Perillae 15 g, Cortex Magnoliae Officinalis 10 g, Bryozoatum 30 g, and Bulbus Fritillariae Cirrhosae 30 g.

*Case 3*. A female, 64 years old, developed recurrent cough and yellow sputum for 40 years. Cough was frequent and sputum was yellow, thin, and in large volume, complicated by chest tightness, wheezing, spontaneous sweating, and poor sleep, but appetite was good, and the patient had a history of chronic gastritis and gastroesophageal reflux. Chest CT: bronchiectases in the lungs were complicated by infection and pulmonary bullous in the lungs. Tongue was light red, coating was thin and greasy, and pulse was thin and slippery. TCM syndrome differentiation was lung and spleen qi deficiency with phlegm-heat.* Treatment rules* are to tonify the spleen and benefit qi, clear the lung heat and reduce phlegm, and tranquilize the mind.* Syndrome differentiation (individualized) decoction* is as follows: Radix Ophiopogonis 10 g, Poria 10 g, Astragalus Astragali 15 g, Rhizoma Fagopyri Cymosi 30 g, Radix Scutellariae 20 g, Herba Violae 30 g, Platycodon grandiflorum 5 g, Semen Coicis 30 g, Radix Asteris 10 g, Inula flower 9 g, Calcined Concha Arcae 30 g, Pericarpium Citri Reticulatae Viride 6 g, Cuttlebone 10 g, Ardisia japonica 30 g, Flos Farfarae 12 g, Poria with hostwood 20 g, Angelica Sinensis 10 g, Spica Prunellae 15 g, and Fructus Tritici Levis 15 g.

*Case 7*. A male, 58 years old, had repeated cough and sputum for more than 30 years, which increased over the past 3 years, and repeated hemoptysis. He had cough and yellow sputum and felt better after expectoration, panting on exertion, general lassitude, chest tightness, occasional chest pain, dry mouth, night sweats, and habitual constipation, but appetite was good. Tongue was red with cracks, coating was thin with yellow color, and the pulse was thin and slippery. Chest CT showed mild bronchiectasis in two lungs. TCM syndrome differentiation is as follows: Qi and yin deficiency with phlegm-heat and dryness in the intestines.* Treatment rule* is To nourish yin and benefit qi, to clear the lung heat and reduce phlegm, moistening the intestine to abduct stagnation.* Syndrome differentiation (individualized) decoction *is as follows: Radix Adenophorae 12 g, Radix Glehniae 12 g, Radix Scutellariae 15 g, Radix Asteris 10 g, Radix Rehmanniae 15 g, Semen Benincasae 20 g, Fructus Ligustri Lucidi 20 g, Poria 15 g, Fructus Trichosanthis 20 g, Herba Violae 30 g, Cortex Magnoliae Officinalis 10 g, Rhizoma Polygoni Cuspidati 15 g, Radix Scrophulariae 15 g, Semen Persicae 10 g, Aloe 4 g, Fructus Mori 15 g, Semen Coicis 15 g, Astragalus Astragali 15 g, Cacumen Biotae 15 g, and Radix Glycyrrhizae 5 g.

*(C) Herbal Preparation and Quality Assurance*. Pieces of traditional Chinese medicinal herbs which had passed quality inspection in line with the national norms [16] were provided by the hospital pharmacy. The decoction of TCM was made according to the literature [17] in the decoction room of our hospital. Pieces of herbs were wrapped in nonwoven cloth bag, soaked in water for 30 min, and decocted 1 time for 60 min in a TCM decocting machine manufactured by Tianjin Sanyan Precision Machinery Ltd. (model: DJQ252). The Chinese herbal decoctions were taken by one decoction a day and divided into 2 doses.

### 2.6. Outcome Measures

The referring physician saw the patients before and after each treatment period and collected data. We asked the patients to identify the symptoms that bother them and a self-administered patient diary or questionnaire was made. The following were the outcome measures.

#### 2.6.1. Primary Outcome: Patient Self-Rated Symptom Score

Patients rated the severity of the symptoms (cough, expectoration, shortness of breath, chest pain, loss of appetite, fatigue, insomnia, and so on) on a 7 point Likert scale [1, 10]. The number of questions must be optimized to ensure that the most important aspects of the patient's problem are examined (usually four to eight items). Every day each patient scored the severity of these problems on the 7 point Likert scales. The higher the score, the more severe the symptom. Taking cough as an example:

On average, in comparison with your usual cough, how severe was the cough?

1. No cough, or as mild as, or milder than they have ever been.
2. Not nearly as severe as usual.
3. Not as severe as usual.
4. As severe as usual.
5. Severer than usual.
6. Very severe, almost as severe as they have ever been.
7. Very severe, as severe as or more severe than they have ever been.

If the participant selected shortness of breath on exertion as a limitation that was important in his or her day-to-day life, we asked the following:

Please indicate how short of breath you have been while on exertion during the past day by choosing one of the following options:

1. Not at all short of breath;
2. A little short of breath;
3. Mildly short of breath;
4. Moderately short of breath;
5. Quite a bit short of breath;
6. Very short of breath;
7. Extremely short of breath.

We consider an improvement of 0.5 points per question corresponds to a noticeable improvement in the patient's well-being. If there are seven questions, a total change of 3.5 or more points is considered clinically important [1, 10]. Thus the mean difference of 0.5 points was defined as the “Minimal Clinically Important Difference (MICD)” for the 7 point scales.

#### 2.6.2. Other Outcomes

*(1) 24 h Sputum Volume*. We measured the 24 h sputum volume and took the mean value for the 3 consecutive days at the beginning and the end of each trial. To ensure the accuracy of the measurement, we asked the patients to spit sputum into a collector with scales from 8:00 am to the next 8:00 am. We used the mean value of the sputum volume for 3 consecutive days as the outcome.

*(2) Safety Outcome*. Blood and urine routine examination, together with liver and kidney function, were determined before and after each N-of-1 trial. We recorded adverse events that occurred and, if necessary, terminated the trial and unblended the code.

*(3) COPD Assessment Test (Chronic Obstructive Pulmonary Disease Assessment Test, CAT).* In recent years, Lee et al. [18] confirmed that the chronic obstructive pulmonary disease assessment test (CAT) was valid and reliable in patients with bronchiectasis. CAT questionnaire is composed of 8 items. Each item has a score ranging from 0 to 5, thereby making the total score range from 0 to 40. Score of 0 represents the best quality of life and 40 does the worst. The MCID (The Minimal Clinically Important Difference) for the CAT has not been established officially, but it was estimated to be around 2 points [19, 20].

### 2.7. Data Analysis

#### 2.7.1. Sample Size Calculation

Estimation of the needed sample size was based on having at least 80% power (*β* = 0.20) to detect a mean difference of 0.5 points (the “Minimal Clinically Important Difference (MICD)) in Patient Self-Rated Symptom Score, which was the main outcome of the study, with significance testing at the *α* = 0.05 level. Standard deviation (SD) was 0.53 based on the data of our study; using a two-sided test, the ratio of the two groups is 1 : 1, with three cross-overs, assuming no period effect or treatment × time interaction, under the given model parameters [21, 22]. We used PASS 11.0 software (NCSS LLC, Kaysville, UT, USA) to calculate the sample size. The result showed that 12 patients would be needed to satisfy the same significance and power requirements. Considering the high drop-out rates of N-of-1 trial (30%), the final sample size was determined to be 16.

#### 2.7.2. Clinical Efficacy Criteria

According to the literatures [1, 23], we made the following standards of clinical efficacy criteria.

(1) The difference in the mean symptom score of at least two pairs out of three pairs is more than 0.5 points. (2) Both the clinician and patient are convinced that one kind of decoction is better and this kind of decoction is confirmed to be the tested drug after unblinding. (3) Acute exacerbations occur in at least two pairs in the second half of the observation period (within 3rd-4th weeks), and the exacerbations are confirmed to occur during the periods of control drug after unblinding. We considered the patient as a “responder” if two of the above three criteria were attained. The clinical efficacy can be assessed in accordance with the above standards for those who did not complete three cycles (pairs) due to acute exacerbations.

As acute exacerbation was included in the clinical efficacy criteria; we can still evaluate the efficacy in a N-of-1 trial if any of the observation periods was terminated due to acute exacerbation.

#### 2.7.3. Statistical Analysis

The values of the outcomes were measured in the last week of each observation period, to avoid the carryover effects of the previously used drug. We took the mean value of the data collected from the last week of each observation period to reduce autocorrelation (i.e., the data are not independent) [10]. The statistical analyses were performed using SAS 9.4 (SAS Institute, Cary NC). If data are normally distributed, we use paired *t* test for single case and mixed effects model for a series of N-of-1 trials as a group. Paired Wilcoxon signed rank tests were conducted to analyze the data with nonnormal distribution. A *P* value of less than 0.05 was considered statistically significant for each test.

### 2.8. Ethics

The trial protocol was approved by the Ethics Committee of Yueyang Hospital, Shanghai University of Traditional Chinese Medicine (number 2013016). Volunteers were recruited through medical lectures and advertisement; volunteer patients were from Shanghai city. The outpatient physicians first screened the subjects by inclusion and exclusion criteria. Based on the guidelines and specifications of the Good Clinical Practice (GCP), we then explained to the subjects in detail about the nature of N-of-1 trials, the possible benefits and harms, and other alternative medications or methods for people who decided not to participate. Whether or not to participate in the study is completely voluntary and participants can withdraw from the study at any time during the study. Finally, the informed consent was obtained.

## 3. Results

### 3.1. The Results of the Preliminary Trial and the Overall Summary of a Series of N-of-1 Trials

**Figure 2:**
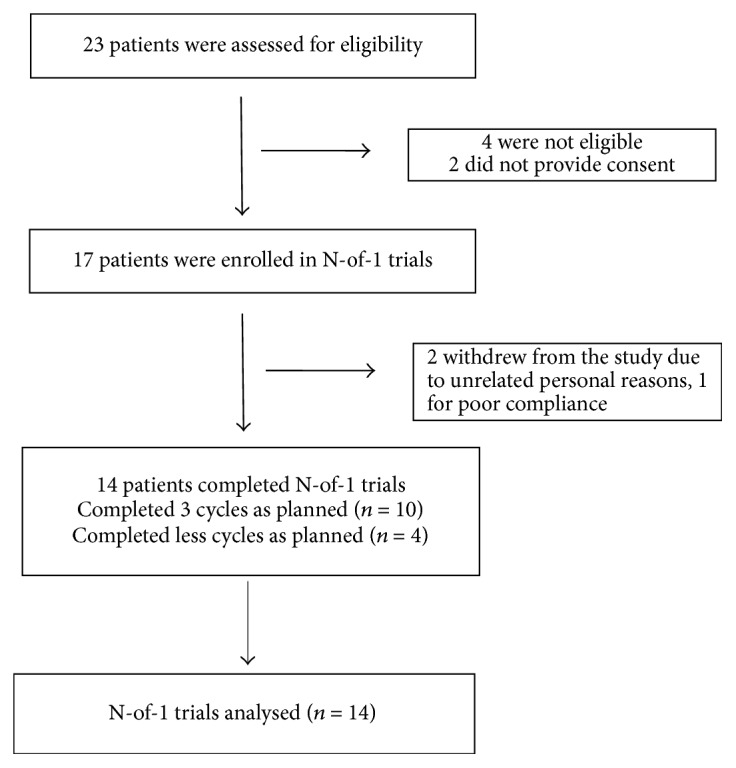
The flowchart of the whole process including recruitment, enrollment, and completion of the study.

We conducted the study in the clinic of the Department of Respiratory Disease, Yueyang Hospital of Integrated Traditional Chinese and Western Medicine, Shanghai University of Traditional Chinese Medicine, from September 2012 to June 2015. Among the nineteen patients meeting the inclusion criteria, seventeen signed the informed consent form and were enrolled in this study (Figure 2). Fourteen cases completed the N-of-1 trial under the supervision of the research group (completion rate 82.35%), as shown in Figure 2. Among the three participants who dropped out of the study, two withdrew during the first round of the trial due to unrelated personal reasons and one for poor compliance. Among the 14 cases who completed the trial, 10 cases had complete data of three cycles; the remaining 4 cases lost the data of some cycles due to acute exacerbation during the trials but contained the intact data of at least 2 observation periods in one cycle (pair).

The 17 patients enrolled in this study attended the open-label preliminary trial to observe the onset time point and the efficacy maintenance time point of syndrome differentiation decoction (test drug). All of them responded. The onset time ranged $8.18\pm 4.86\;(\overset{-}{x}\pm s)$ days and the efficacy maintenance time $9.18\pm 4.36\;(\overset{-}{x}\pm s)$ days. The length of the observation periods in 3 cycles for the 17 participants of N-of-1 trials was determined: both Case 1 and Case 2 took two weeks, Case 3 took three weeks, and the rest of the 14 cases took four weeks.

The 14 cases of patients were roughly divided into qi and yin deficiency syndrome (10 cases) and lung and spleen deficiency syndrome (4 cases), according to TCM syndrome differentiation diagnostic criteria. The baseline data of the 14 patients were listed in Table 1.

### 3.2. The Results of the Individual Data of the N-of-1 Trials

Significant differences (*P* < 0.05) were found in 5 of 14 N-of-1 trials between individualized herbal decoction and control decoction on symptoms score. However, the mean differences of each N-of-1 trial did not reach the standard of clinical significance (>0.5 points) and neither did the 95% CI. Two of the 14 cases were considered as “responder” in accordance with the clinical criteria we drew up (see Section 2.7.2 of this article). Among them case 3 was assessed in accordance with the clinical criteria (1) and (2). Case 5 was assessed according to the clinical criterion (3). Each patient participating in N-of-1 trials was asked to give a global assessment of the effectiveness of the medication and indicate a preference after each treatment pair. Only case 3 and case 5 could distinguish these two decoctions and medication sequence by their effects. After unblinding, it was known as the Individualized decoction. Majority of the participants had no preference to either of the two decoctions. The data in detail were shown in Table 2.

### 3.3. The Results of the Group Data of the Symptom Scores, the 24-Hour Sputum Volume, and the CAT Scores from the 14 N-of-1 Trials

Summarizing the data from each cycle (pair) of a series (14 cases) of N-of-1 trials, we found that individualized decoction was statistically better than control decoction on symptom scores (*P* = 0.002), 24 h sputum volume (*P* = 0.009) and CAT scores (*P* = 0.013). See Table 3. However, the intervention only showed a 0.18 decrease on the mean difference of symptoms severity comparing to the control group. The 95% CI showed that on lower side there was a 0.10 decrease on the mean difference of symptoms severity and on the upper side a 0.25 decrease on the mean difference of symptoms severity. All of which did not reach the standard of clinical significance (0.5 points). Similarly, the intervention only showed a 1.95 decrease on the mean difference of CAT scores comparing to the control group, failing to reach the estimated MICD of CAT scores (2 points).

**Figure 3:**
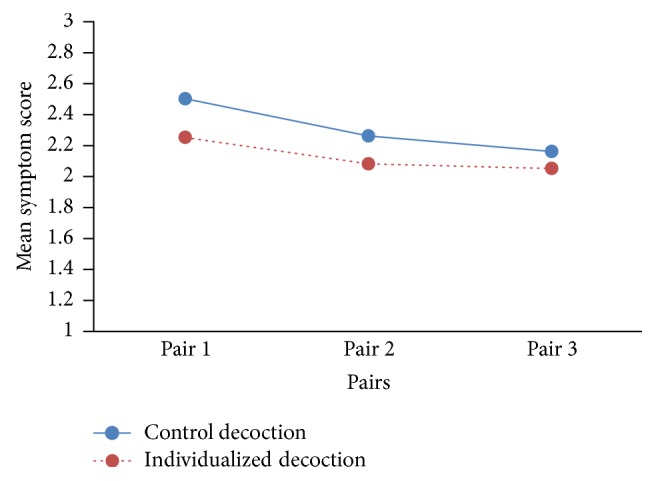
The comparison of the mean symptom scores between the individualized decoction and control decoction in the first, second, and third pairs of a series (14 cases) of N-of-1 trials.

Using symptom scores as the index, we found that the mean scores of the individualized decoction were lower than those of the control decoction in the first, second, and third pairs of a series (14 cases) of N-of-1 trials. However, the gap between the two had a diminishing trend from first to third pairs (Figure 3).

### 3.4. Safety Outcome

No severe adverse events occurred during the whole study. The blood and urine examination including liver and kidney function was normal before and after each N-of-1 trial. Only a case of minor gastrointestinal tract reaction (anorexia) was documented, which was alleviated after adding Radix Saussureae 10 g and Fructus Amomi 6 g to regulate qi-flowing for harmonizing stomach. This minor adverse event did not affect the process of the study and was determined after unblinding to have been caused by syndrome differentiation (individualized) decoction.

## 4. Discussion

### 4.1. Summary of the Results in This Study

After the clinical trials all the patients claimed to have improved to varying degrees on symptoms, sputum volume, and quality of life, and their compliance with this series of trials is high (completion rate 82.35%). The results of the individual data of the N-of-1 trials showed that significant differences (*P* < 0.05) were found in 5 of 14 N-of-1 trials between individualized herbal decoction and control decoction on symptoms score. However, each of these cases did not reach the standard of clinical significance based on mean differences (>0.5 points) or 95% CI. Two of the 14 cases were considered as “responder” according to the clinical criteria. The 14 N-of-1 trials as a group showed that for individualized decoction, compared to the control decoction, there were significant decreases in symptom scores (*P* = 0.002), 24 h sputum volume (*P* = 0.009), and CAT scores (*P* = 0.013). However, the mean differences and 95% confidence interval between the two decoctions on the primary outcome were 0.18 and 0.10, 0.25. According to the MICD of symptoms scores (0.5 points) (see Section 2.6.1 of this article), the difference between the two decoctions was considered statistically significant, but not clinically important. Similarly, the mean difference of CAT scores between the two groups was not considered clinically important as it failed to reach the estimated MICD of CAT scores (2 points). No major adverse events were reported.

### 4.2. Comparing Findings with Other Studies

The investigation of the individualized treatment of traditional Chinese medicine by use of N-of-1 trials is still in the exploratory stage. There have been a few research papers of N-of-1 trials on the effect of traditional Chinese medicine [24–26]. These researches showed that N of-1 trials were feasible and reflected the advantage of individualized treatment of TCM. However, the methodology needs to be improved. One of the trials did not use blinding; no method for determining the washout period was reported in any of the trials.

In this study, we tried to evaluate the washout period through preliminary trials and used two different Chinese medicine decoctions in a series of N-of-1 trials with blinding of patient, care provider, and outcome assessor. Bronchiectasis stabilization decoction used as the control was more easily accepted by the patients than placebo, which has been shown to be effective as a basic formula when integrated with syndrome differentiation in a previous randomized controlled trial [9]. Furthermore, the comparison between the control decoction (bronchiectasis stabilization decoction) and the individualized decoction may be the best way to reflect the individualized treatment of TCM. In addition, we have explored the combined analysis of individual data and group data, the combination of statistical analysis, and clinical efficacy criteria, in a series of N-of-1 trials in this study.

### 4.3. Strengths and Limitations

The decoction of Traditional Chinese medicinal herbs is still the main form of TCM clinical practice, so the comparison between the two different Chinese medicine decoctions in N-of-1 trials is closer to clinical practice. It is easier to be accepted by patients than placebo control. In our study the two TCM decoctions could be similar in appearance and size, but the slight difference in taste and smell may still exist. To deal with the situation, we told the participants that both of the two decoctions could be effective regardless of the taste and smell. The implementation of this blinding method was generally successful, as most participants (12/14) were not aware which type of formula they were assigned to and did not show preference towards certain decoction. The objectivity of efficacy evaluation of both doctors and patients improved compared to previous trials of traditional Chinese medicine without blinding [9, 25].

Besides possible limitations to blinding due to learning perceptible differences between decoctions such as smell and taste, we identified some other limitations: First, the syndrome differentiation (individualized) decoction theoretically should be superior to the control (fixed) decoction. However, due to the lack of statistical power, the data of a single N-of-1 trial (only 3 cycles) might not be sufficient to show significant difference between intervention and control arms. Second, the nature of traditional Chinese medicine might not meet with a certain requirement of the classic N-of-1 trials perfectly: The treatment should have a rapid onset and stop acting soon after it is discontinued [1, 10]. One of our results showed that the gap between the mean symptom scores of the individualized decoction and control decoction had a diminishing trend from first to third pairs in a series (14 cases) of N-of-1 trials (Figure 3), suggesting the possibility of “carryover effects.” This point of view is similar to the study of Yuhong et al. [27] who reported a study of N-of-1 RCTs testing the effectiveness of Liuwei Dihuang decoction (LDD) for kidney-yin deficiency syndrome. Each period consisted of 4-week LDD and 4-week placebo in random order in the study. They discussed that a limitation of the trial was washout period which had not been fully considered, which resulted in residual effects of traditional Chinese medicine interfering with the differences between LDD and placebo.

### 4.4. Implications for Clinical Practice and Future Research

*(1) Exploring the Establishment of the Clinical Effect Evaluation Method Which Is Consistent with the Characteristics of TCM Individual Diagnosis and Treatment*. As mentioned earlier, the idea of individualized treatment represented by N-of-1 trials coincides with the principle of syndrome differentiation of TCM. The establishment of a reliable clinical effectiveness evaluation method based on evidence-based medicine is essential to the further development and internationalization of TCM. N-of-1 trials might be a method to evaluate this basic therapeutic principle of individualization established through thousands of years of clinical practice of TCM.

As the differences between the individualized decoction and control decoction in this study were considered statistically significant but not clinically important, the results showed the tendency that individualized decoction was better than the control (standard) decoction. To improve the efficiency of this study design, we suggest (a) further extending the observation period, or increasing the numbers of rotation to 4-5 cycles (pairs). This means that the whole duration of a single N-of-1 trial would last more than half a year. Furthermore, some uncertain factors interfering with the completion of the trial will increase. (b) X. Chen and P. Chen [28] tried to provide a practical guidance for the analysis of N-of-1 trials by comparing four commonly used models (paired *t*-test, mixed effects model of difference, mixed effects model, and meta-analysis of summary data). They concluded that mixed effects model provided an alternative when there was carryover effect for normally distributed data of N-of-1 trials. If the factor of carryover effect is added to statistical models, the efficiency of the data analyses of N-of-1 trials for TCM may be improved. (c) Conduct placebo-controlled N-of-1 trials which have a higher detection sensitivity; the ability to detect absolute efficacy and safety; and better research of carryover effects.

*(2) The Combined Analysis of Individual Data and Group Data*. While stressing the individualized treatment in N-of-1 trials, we would suggest that aggregated data of a series of N-of-1 trials can reflect the generality of the results in the study. Due to their crossover design, aggregated N-of-1 trials have a smaller required sample size than their conventional parallel arm RCT counterparts for equivalent levels of statistical power and are better at controlling for confounding factors [29]. We successfully used mixed effects model [28] for the statistical analysis of a series of N-of-1 trials as a group. There are a number of statistical methods proposed for the analysis of N-of-1 trials. In recent years, hierarchical Bayesian statistical methods have gotten ever-broader use in this field. Senior et al. [30] summarized that the advantages of a Bayesian approach over normal frequentist statistical methods allow (a) both individual and aggregate analyses to be simultaneously and coherently undertaken–even when the number of completed cycles between patients is variable, (b) they exploit and accommodate natural hierarchies and serial correlations within the study (such as clustering by physician, setting, or location), (c) the outcome variable of interest can take any functional form, (d) confounding variables can easily be introduced, (e) they naturally allow the incorporation of any relevant trial information that may be sourced from elsewhere, and (f) they produce sensible estimates and confidence intervals. Thus hierarchical Bayesian statistical methods may be used for the research of TCM in the future.

*(3) About the Clinical Efficacy Criteria*. In a classic study of N-of-1 trials completed by Guyatt et al. [1], of 70 N-of-l trials begun, 50 (71%) provided a definite clinical or statistical answer after the trial. However, when using rigorous statistical criteria for a definite answer, such an answer was attained in only 43% of the trials. So Guyatt wrote in his representative publication introducing N-of-1 trials [8] that “the use of N-of-1 trials to improve patient care does not depend on the statistical analysis of the results.” As a single N-of-1 trial only has limited pairs resulting in low statistical power, it is difficult to reach a conclusion merely depending on the statistical results. While statistical analysis can be helpful for interpreting the results, the use of clinical efficacy criteria may play an important role in the evaluation of N-of-1 trials [1, 10, 23]. We drew up the clinical efficacy criterion (1) based on the results of symptom scores on a 7-point Likert scale [1, 10]. We also drew up the clinical efficacy criterion (3) of the study by adding the factor of acute exacerbations (see Section 2.7.2 of this article). Acute exacerbation may occur in stable bronchiectasis. There were a few patients who experienced acute exacerbations in this study, leading to temporary discontinuation of the trial. We found that the frequency of acute exacerbation of bronchiectasis decreased significantly in the periods of individualized decoction compared to control decoction (data not shown). How to improve the objectivity and reliability of clinical efficacy criteria deserves further exploring.

The ultimate stakeholder in medical decision making is the patient. The most attractive aspect of N-of-1 trials is that a presumptively or potentially more effective prescription or treatment can be screened through the comparison of the two treatments for the individual patient. By repeating more N-of-1 trials on the same patient, we may constantly improve the effect of drugs (or decoctions) for each individual. That is the ultimate goal for the research of individual treatment (syndrome differentiation) by TCM.

In summary, this study showed a trend towards the beneficial effects of individualized herbal decoction comparing to the standard herbal decoction through a series of N-of-1 trials. Optimizing the combined analysis of individual data and group data, the combination of statistical analysis and clinical efficacy criteria, and the improvement of statistical models may contribute to setting up the clinical efficacy evaluation method in line with the characteristics of TCM individual diagnosis and treatment.

## Figures and Tables

**Table 1:** Demographic and clinical characteristics of the 14 completers/partial-completers.

Gender (male/female)	14 (3/11)
Age in years—mean (minimum, maximum)	64.14 (50, 72)
Bronchiectasis in chest CT (unilateral/bilateral)	(3/11)
TCM syndrome differentiation (lung and spleen deficiency syndrome/qi and yin deficiency syndrome)	(4/10)
Concomitant medication (Yes/No)	(2/12)
Baseline of the outcomes	Mean (SD)
Symptom scores	2.76 (0.136)
24-hour sputum volume	24.45 (5.89)
CAT scores	15.27 (1.57)

**Table 2:** The mean value of patient self-reported symptom scores (individualized decoction versus control decoction) of each N-of-1 trial.

Number of case	Control decoction			Individualized decoction			Mean difference (95% CI)	*P*-value	Preference to the two decoctions
	Cycle 1	cycle 2	cycle 3	Cycle 1	cycle 2	cycle 3			
Case 1	1.46	1.41	1.11	1.44	1.49	1.11	−0.02 (−0.16, 0.11)	0.54	No
Case 2	3.07	-	2.85	3.00	-	3.11	*∗*	*∗*	No
Case 3^▲^	3.33	3.49	3.19	2.80	2.84	3.37	0.34 (−0.78, 1.45)	0.32	Individualized decoction
Case 4	2.06	1.68	1.56	1.91	1.62	1.67	0.03 (−0.29, 0.36)	0.71	No
Case 5^▲^	-	-	2.27	-	-	2.23	*∗*	*∗*	Individualized decoction
Case 6	1.67	1.63	1.83	1.53	1.71	1.83	0.02 (−0.25, 0.29)	0.78	No
Case 7	3.13	2.63	2.70	2.66	2.34	2.23	0.41 (0.15, 0.67)	0.02	No
Case 8	3.40	2.52	2.38	2.83	2.40	-	*∗*	*∗*	No
Case 9	1.89	2.06	1.88	1.79	1.91	2.00	0.04 (−0.32, 0.41)	0.66	No
Case 10	2.20	2.10	2.07	2.07	1.80	1.83	0.22 (0.02, 0.43)	0.04	No
Case 11	2.68	2.28	2.12	2.54	2.20	2.01	0.11 (0.03, 0.19)	0.03	No
Case 12	3.46	2.89	2.79	3.17	2.74	2.49	0.25 (0.03, 0.47)	0.04	No
Case 13	2.48	2.14	1.76	2.10	1.81	1.39	0.36 (0.30, 0.42)	0.001	No
Case 14	1.69	-	1.71	1.36	-	1.36	*∗*	*∗*	No

^−^Data were not included in the statistical analysis due to an acute exacerbation during the cycle. ^*∗*^No individual statistic analysis due to incomplete data. ^▲^Considered as “responder” according to the clinical criteria.

**Table 3:** The comparison between the individualized decoction and control decoction based on the group data of the symptom scores, the 24-hour sputum volume, and the CAT scores from the 14 N-of-1 trials.

	*n*	Control decoction Mean (SD)	Individualized decoction Mean (SD)	Mean difference (95% CI)	*P* value
Symptom scores	37	2.30 ± 0.65	2.13 ± 0.58	0.18 (0.10, 0.25)	0.002^▲^
24 h sputum volume (ml)	37	(7.00, 17.49, 32.41)^#^	(5.50, 15.80, 34.84)^#^	*※*	0.009^#^
CAT scores	29	11.64 ± 5.59	9.69 ± 4.89	1.95 (1.04, 2.86)	0.013^▲^

^▲^These values were the results of mixed effects model. ^#^These data with nonnormal distribution were expressed in median (interquartile spacing) and calculated with paired Wilcoxon signed rank test. ^*※*^Not available.
